# The Protective Effects of a Modified Xiaohua Funing Decoction against Acute Liver Failure in Mice Induced by D-Gal and LPS

**DOI:** 10.1155/2022/6611563

**Published:** 2022-01-13

**Authors:** Jindong Zhao, Lili Liu, Ling Xin, Yunxia Lu, Xiaojun Yang, Yong Hou, Mei Shi, Sha Han, Hao Zhou, Yonghua Liu, Zhaohui Fang, Yan Li, Guoliang Zhang

**Affiliations:** ^1^Graduate School, Anhui University of Chinese Medicine, Hefei 230012, China; ^2^Department of Endocrinology, The First Affiliated Hospital of Anhui University of Chinese Medicine, Hefei 230031, China; ^3^Department of Infectious Disease, The First Affiliated Hospital of Anhui University of Chinese Medicine, Hefei 230031, China; ^4^Department of Information, The First Affiliated Hospital of Anhui University of Chinese Medicine, Hefei 230031, China; ^5^Department of Biochemistry and Molecular Biology, Anhui Medical University, Hefei 230032, China

## Abstract

**Objective:**

The aim of this study was to evaluate the effects of a modified Xiaohua Funing decoction (Xfd) on acute liver failure (ALF) and determine whether the protective mechanisms are related to alterations in the gut microbiota.

**Methods:**

An animal model of ALF was induced by intraperitoneal injection of D-galactosamine (D-Gal, 0.5 g/kg) and lipopolysaccharide (LPS, 100 *μ*g/kg). Male BALB/*c* mice were randomly divided into the following 4 groups: the control group (saline, Con), model group (D-Gal/LPS, Mod), silymarin pretreatment group (200 mg/kg, Sil), and modified Xfd pretreatment group (650 mg/kg, Xfd). The Sil and Xfd groups received the respective intervention orally for 14 days and 2 h before D-Gal/LPS treatment. The liver injury markers included alanine aminotransferase (ALT) and aspartate aminotransferase (AST) levels and liver histology. 16S rRNA gene sequencing was performed to assess the effects on the caecum content.

**Results:**

D-Gal/LPS treatment caused severe ALF, illustrating that the ALF model was successfully established. The administration of Sil and Xfd greatly reduced the serum ALT and AST levels and improved the pathological signs of liver injury. However, no significant difference was found between the two groups. In contrast to the Mod group, the Sil and Xfd groups showed a shift toward the Con group in terms of the gut microbiota structure. The abundances of Firmicutes and Bacteroidetes and the Bacteroidetes/Firmicutes ratio in the Mod group significantly differed from those in the Con group. The Sil and Xfd groups showed restoration of the disordered microbiota. Significantly increased relative abundances of Lachnospiraceae_NK4A136_group and Candidatus_Saccharimonas and a markedly decreased Muribaculaceae abundance were found in the Sil and Xfd mice compared with those in the Mod mice (*P* < 0.01, *P* < 0.05). Interestingly, a negative correlation was observed between the abundances of the gut microbiota constituents, specifically Clostridia_UCG-014, and ALT and AST levels.

**Conclusion:**

In summary, our results indicate that Xfd may protect the liver and modify the gut microbiota in ALF mice.

## 1. Introduction

Acute liver failure (ALF) caused by extensive hepatocyte necrosis is a fatal clinical syndrome and has been well characterized. However, the underlying mechanisms are not fully understood [1]. Studies have demonstrated that alterations in the gut microbiota play a crucial role in the pathogenesis of liver diseases [2, 3]. During the early stage of ALF, the systemic and intestinal immune systems are suppressed, which leads to a rapid loss of normal liver function, multiple organ failure, and even death [4]. Despite advances in medical therapies, effective clinical ALF treatment is lacking [5, 6].

In China, Chinese herbal medicine (CHM) therapy for ALF has been widely used. Several clinical trials have shown that CHM can provide ALF patients with important alternative and complementary therapy benefits, such as improvement in systemic and liver recovery, regulation of inflammatory mediators, and induction of antiapoptotic genes [7–9]. Modified Xiaohua Funing decoction (Xfd) was developed by a nationally celebrated expert in traditional Chinese medicine (TCM), Jingshi Xu. Here, we report that Xfd includes *Bambusae Caulis in Taenias, Bupleuri Radix, Pinelliae Rhizoma Praeparatum cum Zingibere et Alumine, Aurantii Fructus, Setariae Fructus Germinatus, Paeoniae Radix Alba, Schisandrae Fructus Chinensis, Salviae Miltiorrhizae Radix et Rhizoma, Glehniae Radix, Citri Reticulatae Pericarpium,* and *Mume Fructus*. For over five decades, Xfd has been known to have a significant impact on liver diseases. Our previous clinical studies suggested that Xfd improved liver function and other symptoms, such as alanine aminotransferase (ALT), aspartate aminotransferase (AST), total protein, total bilirubin, and alkaline phosphatase levels; lateral thorax pain; stomach pain; loss of appetite; abdominal distension; loose stools; and jaundice. Xfd not only is effective but also has no other adverse effects, such as induction of kidney failure and diarrhea [10, 11]. Several protective mechanisms of Xfd have been investigated based on the inflammatory response and the extracellular regulated protein kinase (ERK) signaling pathway [12].

The classical liver injury model induced by D-galactosamine (D-Gal)/lipopolysaccharide (LPS) has been used to evaluate abnormal liver function, regulation of metabolic disorders, and variations in the gut microbiota [13, 14]. This mouse experimental model mimics clinically observed ALF [15]. The present study aims to investigate the effects of Xfd on gut microbiota and liver function in the context of ALF.

## 2. Materials and Methods

### 2.1. Materials and Reagents

Xfd contains 11 medicinal components, namely, *Bambusae Caulis in Taenias, Bupleuri Radix, Pinelliae Rhizoma Praeparatum cum Zingibere et Alumine, Aurantii Fructus, Setariae Fructus Germinatus, Paeoniae Radix Alba, Schisandrae Fructus Chinensis, Salviae Miltiorrhizae Radix et Rhizoma, Glehniae Radix, Citri Reticulatae Pericarpium,* and *Mume Fructus*, at a ratio of 15 : 10 : 10 : 15 : 30 : 20 : 10 : 12 : 15 : 12 : 10. Xfd has been authenticated and standardized based on marker compounds according to the Chinese Pharmacopoeia 2020 [16]. A dose of 159 g of herbs was decocted with boiling water and concentrated with a vacuum rotary evaporator until crude aqueous extract. Silymarin was purchased from Macklin Biochemical Co., Inc. (Shanghai, China). Its purity was greater than 80%. LPS (from *Escherichia coli*, 055:B5) and D-Gal were obtained from Sigma-Aldrich Chemical Co. (St. Louis, MO, USA). ALT and AST assay kits were provided by Nanjing Jiancheng Bioengineering Institute (Nanjing, China). A paraformaldehyde solution, acetonitrile, and formic acid were acquired from Macklin Biochemical Co., Inc. (Shanghai, China).

### 2.2. Animals and Treatment

Thirty-two male BALB/*c* mice (7 weeks old, 20–22 g) were obtained from the Shandong Laboratory Animal Center (Jinan, China; certificate number SCXK-20190003). The animals were acclimated in a specific pathogen-free animal laboratory at 22 ± 2°C with 50–70% relative humidity under a 12/12 h light/dark cycle. The mice were fed standard laboratory chow and given water ad libitum.

The mice were randomly divided into 4 groups (*n* = 8). The control group (Con) and model group (Mod) received the same volume of saline intragastrically once daily, the pretreatment silymarin group (Sil) received 200 mg/kg silymarin intragastrically once daily, and the pretreatment Xfd group (Xfd) received 650 mg/kg Xfd intragastrically once daily. The pretreatment course was 14 days. The bodyweight of the mice was measured every week.

Two hours after the last intragastric treatment, the mice in the Mod, Sil, and Xfd groups were treated with D-Gal (0.5 g/kg)/LPS (100 *μ*g/kg) via intraperitoneal injection to induce ALF; D-Gal and LPS were freshly dissolved  in saline. The mice in the control group were treated with the same volume of saline and with the same procedures. The experimental design and mouse handling procedures were in accordance with the guidelines for the care and use of laboratory animals.

### 2.3. Sample Collection and Analysis

Eight hours after treatment, the mice were killed by retroorbital bleeding, and blood samples, liver tissues, and cecal contents were collected.

#### 2.3.1. Assessment of ALF

The blood was centrifuged for 10 min at 14,000 rpm at 4°C. The supernatant was stored at −80°C until biochemical analysis. The ALT and AST levels were determined using an automatic biochemical analyzer (AU 7600, HITACHI, Japan).

#### 2.3.2. Histological Evaluation

The liver tissue was weighed, washed with saline, and immediately placed into a tube containing 4% paraformaldehyde solution. The liver tissues were washed with phosphate-buffered saline (PBS), embedded in paraffin wax, sliced into 5 *μ*m sections, and stained with hematoxylin-eosin (HE). The sections were analyzed under a Zeiss Axioskop 2 Plus upright light microscope equipped with a camera (Zeiss, Oberkochen, Germany).

#### 2.3.3. 16S rRNA Sequencing

The cecal contents were collected in sterile tubes and stored at −80°C until gut microbiota analysis. Total bacterial DNA was extracted using a TIANamp Stool DNA Kit (Tiangen Biotech Co., Ltd., Beijing, China) following the manufacturer's instructions. The 16S rRNA gene in the DNA samples was amplified using the conventional barcoded universal bacterial primers F338/R806 targeting the V3–V4 region, which were selected to analyze the taxonomic composition of the gut microbiota. The concentrations of the PCR products were determined using an Illumina HiSeq platform (Illumina, SD, USA) for paired-end reads with 2 × 300 bp (468 bp) sequencing. The trimmed sequences were uploaded to the Quantitative Insights Into Microbial Ecology (QIIME, v1.8.0) and *R* packages (v3.3.1) [17, 18]. The acquired data were produced by Majorbio Bio-pharm Technology Co., Ltd. (Shanghai, China).

The number of shared and unique operational taxonomic units (OTUs) and taxonomic classifications were determined by BLAST searching the sequences against the Greengenes database at the 97% nucleotide similarity level [19]. The *α* diversity was represented according to the Shannon, Simpson, ACE, and Chao indexes. The taxonomy-based analyses were visualized based on GraPhlAn. The data were visualized via principal component analysis (PCA) [20].

### 2.4. Statistical Analysis

All data are expressed as the mean ± SD and were subjected to one-way analysis of variance (ANOVA), Fisher's least significant difference (LSD) test, or Spearman's correlation test using SPSS 23.0 (SPSS Inc., IBM, USA). The PCA score plots based on phylogenetic statistical analysis methods indicated clustering of the gut microbiota within groups. The Wilcoxon rank-sum test was used to compare the group data of two samples. *P* < 0.05 was considered statistically significant. The results were graphically visualized using GraphPad Prism (v6.0, GraphPad Software, USA), and correlation heatmaps were generated using *R* software (v3.3.1).

## 3. Results

In a preliminary experiment, it was observed that the mice began to die 10 h after D-Gal (0.5 g/kg)/LPS injection; thus, to ensure that no mice died during the experiment, we chose to perform euthanasia at 8 h.

### 3.1. Mouse Body Weight

**Figure 1:**
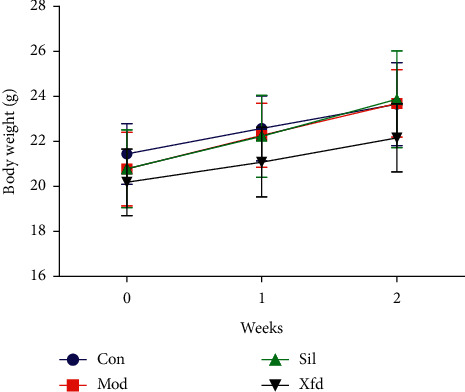
Comparison of body weights.

There was a gradual increase in body weight in all groups. In week 2, the body weights of the mice were not significantly increased in any of the four groups (Figure 1).

### 3.2. ALF Attenuation

**Figure 2:**
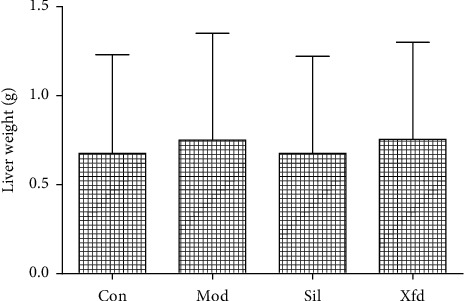
Comparison of liver weights and appearance.

**Figure 3:**
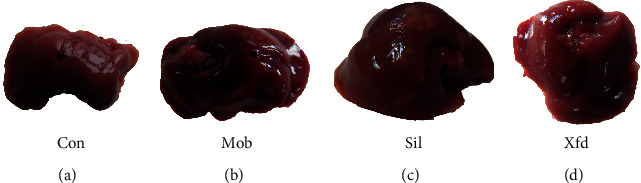
Representative gross liver appearance. (a) Con. (b) Mod. (c) Sil. (d) Xfd.

**Figure 4:**
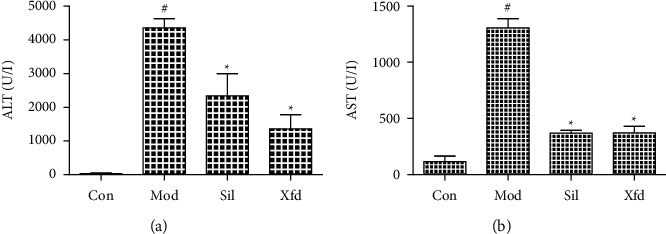
Comparison of ALT and AST levels (a) ALT. (b) AST. *Note.* The values are expressed as the mean ± SD. ^#^*P* < 0.01, compared with Con; ^★^*P* < 0.01, compared with Mod.

The liver weight of the mice did not significantly differ among the four groups (Figure 2). The livers of the control group mice had a light-red smooth surface and a soft texture. In contrast, D-Gal/LPS treatment caused severe liver failure. The livers of the Mod mice were dark red and exhibited coagulation, necrosis, and a slightly hard texture. Sil and Xfd reduced the ALF changes, according to visual observations (Figure 3). The serum ALT and AST levels in the Mod mice were much higher than those in the Con mice. Compared with the Mod group, the Sil and Xfd groups showed remarkably reduced ALT and AST levels, and the Xfd mice showed no significant decreases in ALT and AST levels compared with those in the Sil mice (Figure 4).

**Figure 5:**
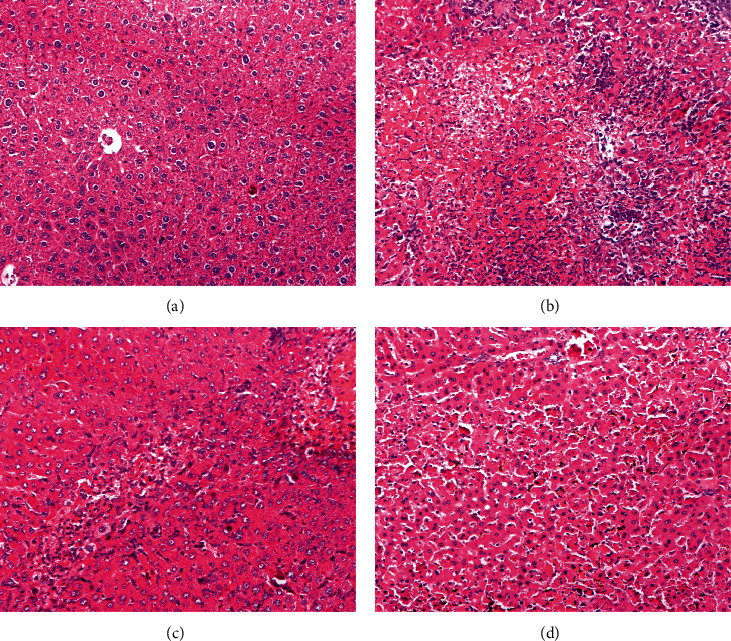
Representative histopathological changes in the liver. *Note.* Original magnification, 200×. (a) Con group. (b) Mod group. (c) Sil group. (d) Xfd group.

The livers of the Con mice had clear liver lobules, and no degeneration or necrosis of liver cells was observed. In contrast, D-Gal/LPS treatment caused severe liver failure. The livers of the Mod mice had a large amount of hepatocyte degeneration, mainly cellular edema. Nuclear lysis, necrosis, inflammatory cell infiltration, massive bleeding, venous congestion and expansion, and no cell proliferation were observed. Compared with the Mod group, the Sil and Xfd groups showed reduced hepatocyte necrosis, bleeding, and inflammation, and the ALF changes were significantly improved (Figure 5).

### 3.3. Community Structure of the Gut Microbiota

**Figure 6:**
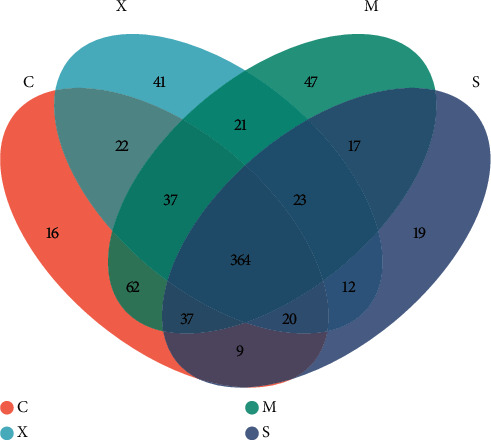
Comparison of OTUs.

To characterize the composition of the gut microbiota, 1,542,312 cleaned sequences were generated from 32 samples. In total, 567, 608, 501, and 540 OTUs were found in the Con, Mod, Sil, and Xfd groups, respectively. In total, 747 operational OTUs were obtained. Moreover, there were 364 common OTUs across the four groups, and 16, 47, 19, and 41 specific OTUs were found in the Con, Mod, Sil, and Xfd groups, respectively (Figure 6).

**Figure 7:**
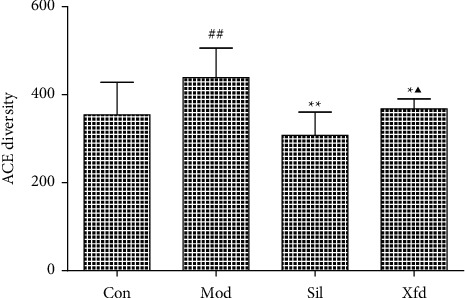
Comparison of the ACE diversity index. *Note.* The values are expressed as the mean ± SD; ^##^*P* < 0.01, compared with the Con group; ^★^*P* < 0.05 and ^★★^*P* < 0.05, compared with the Mod group; ^▲^*P* < 0.05, compared with the Sil group; C: Con group; M: Mod group; S: Sil group; X : Xfd group.

The Shannon, Simpson, ACE, and Chao indexes were analyzed as *α* diversity metrics. There was an obvious increase in the ACE- and Chao-based diversity (*P* < 0.05) following Sil or Xfd treatment (Figure 7). There were no observed differences in the Shannon (*P*=0.055) and Simpson (*P*=0.217) indexes.

**Figure 8:**
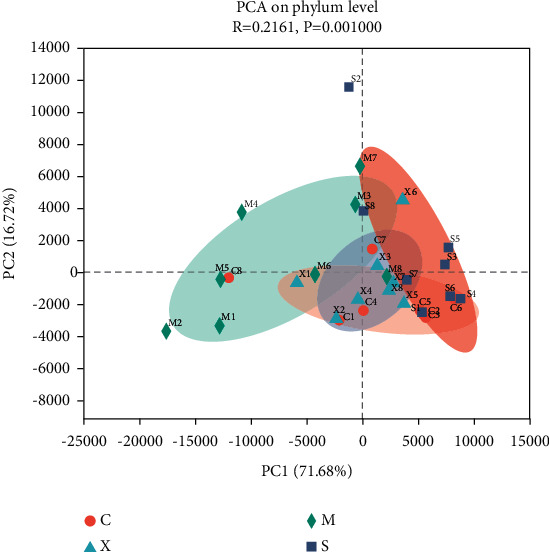
Comparison of the gut microbiota structure using PCA. *Note.* Different colors represent sample groups in different environments; C: Con group; M: Mod group; S: Sil group; X : Xfd group.

**Figure 9:**
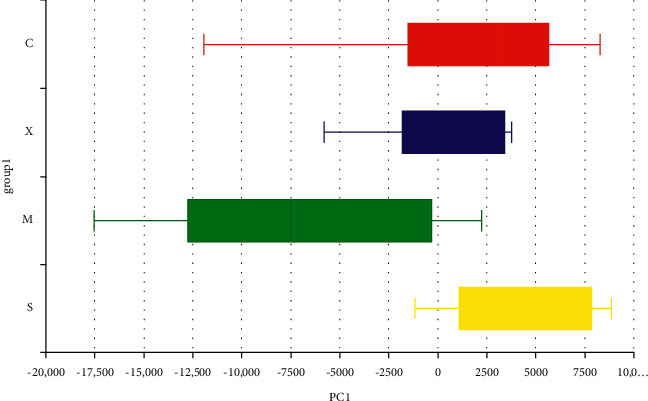
Comparison of the gut microbiota structure along PC1 using PCA. *Note.* The box plot in the figure represents the dispersion of different groups of samples on the PC1 axis; C: Con group; M: Mod group; S: Sil group; X : Xfd group.

The PCA of both unweighted and weighted UniFrac distances showed that the first principal component (PC1) accounted for 71.68% and the second principal component (PC2) accounted for 16% of the variation. PCA also showed a reversal of the changes that occurred in the gut microbiota in response to the Sil and Xfd treatments, especially an obvious shift along PC1 (Figures 8 and 9).

### 3.4. Modulation of the Key Gut Microbiota Community Members

**Figure 10:**
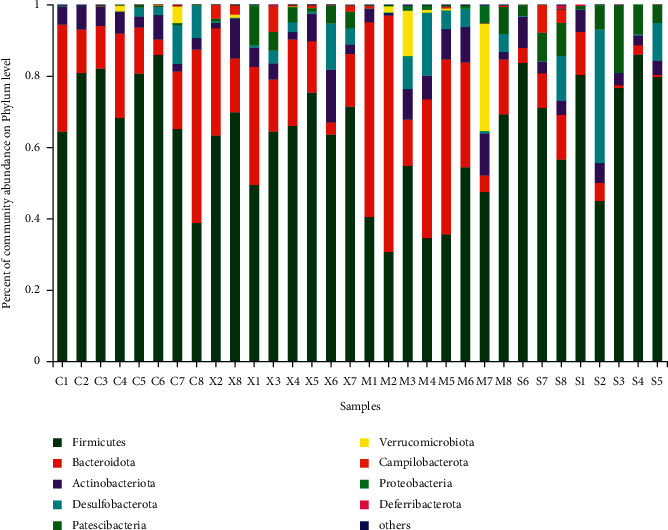
Comparison of the abundances of gut bacterial phyla.

**Figure 11:**
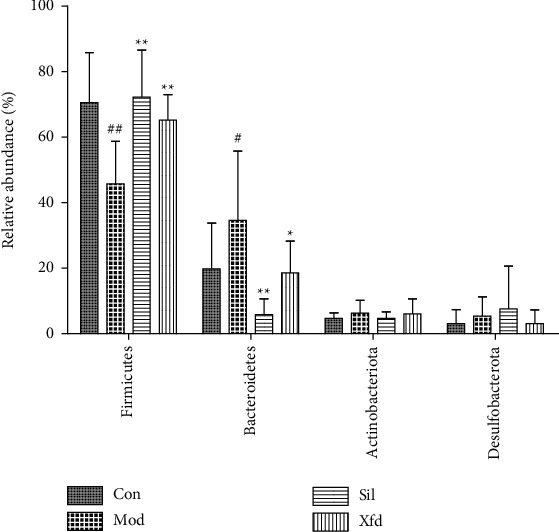
Comparison of the relative abundances of gut bacterial phyla. *Note.* ##*P* < 0.01 versus the Con group; ^★^*P* < 0.05 and ^★★^*P* < 0.01 versus the Mod group.

**Figure 12:**
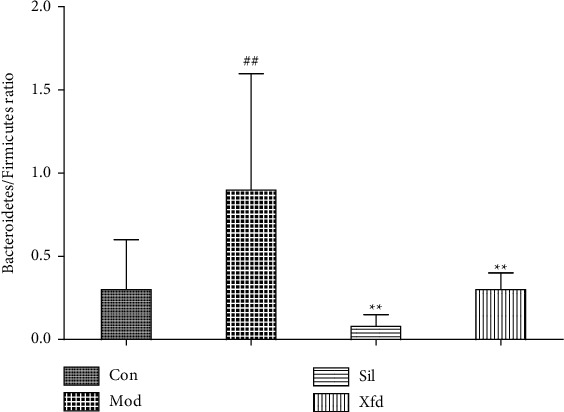
Comparison of the Bacteroidetes/Firmicutes ratio. *Note.*^##^*P* < 0.01 versus the Con group; ^★★^*P* < 0.01 versus the Mod group.

The dominant gut bacteria belonged to the phyla Firmicutes and Bacteroidetes. The composition of each sample is shown in detail (Figure 10). The abundance of Firmicutes in the Mod group (45.85 ± 13.00%) was significantly lower than that in the Con group (70.67 ± 15.34%) (*P* < 0.01). The proportion of Firmicutes showed an increasing trend in the Sil and Xfd groups (72.30 ± 14.30%, 65.37 ± 7.74%) compared with that in the Mod groups (*P* < 0.01, *P* < 0.05). The abundance of Bacteroidetes in the Mod group (34.62 ± 21.23%) was significantly higher than that in the Con group (19.95 ± 13.92%) (*P* < 0.05). The proportion of Bacteroidetes showed a decreasing trend in the Sil and Xfd groups (5.90 ± 4.88%, 18.68 ± 9.71%) compared with that in the Mod group (*P* < 0.01, *P* < 0.05). The gut microbiota of the Mod group mice was characterized by an increased Bacteroidetes/Firmicutes ratio (0.90 ± 0.71), and the ratio showed significant differences, with lower proportions in the Sil and Xfd group mice (0.08 ± 0.07, 0.30 ± 0.19, respectively) (*P* < 0.05, *P* < 0.01). There was no statistically significant difference in the abundances of Firmicutes and Bacteroidetes or the Bacteroidetes/Firmicutes ratio between the Sil and Xfd groups (*P* < 0.05). There were no significant differences in the Actinobacteriota and Desulfobacterota abundances among the four groups (Figures 10–12).

**Figure 13:**
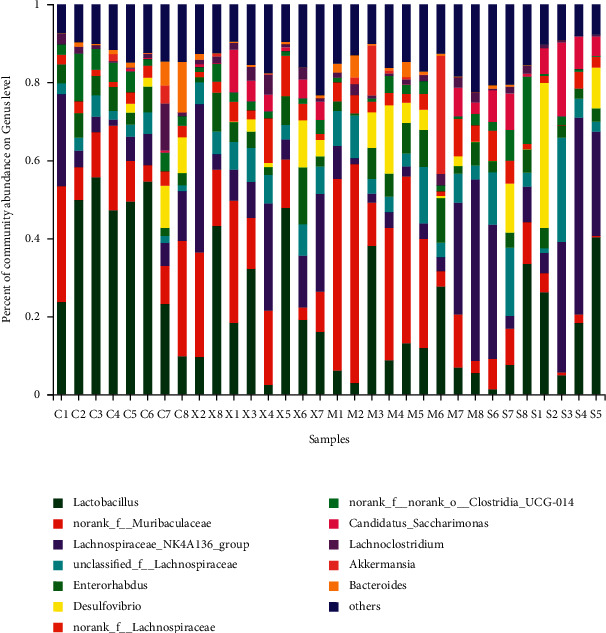
Comparison of abundances among gut bacterial genera.

**Figure 14:**
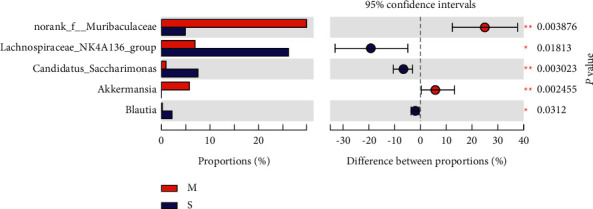
Comparison of the relative abundances of gut bacterial genera between the Mod and Sil groups. *Note.*^★^*P* < 0.05 and ^★★^*P* < 0.01 versus the Mod group; M: Mod group; S: Sil group.

**Figure 15:**
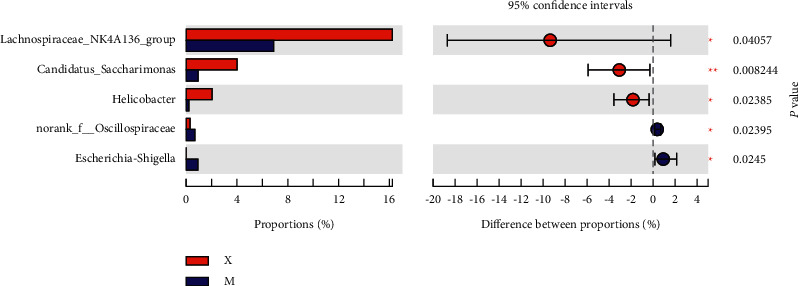
Comparison of the relative abundances of gut bacterial genera between the Mod and Xfd groups. *Note.*^★^*P* < 0.05 and ^★★^*P* < 0.01 versus the Mod group; M: Mod group; X: Xfd group.

**Figure 16:**
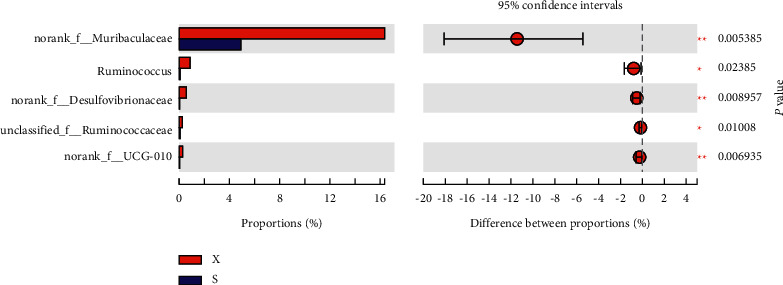
Comparison of the relative abundances of gut bacterial genera between the Sil and Xfd groups. *Note.*^★^*P* < 0.05 and ^★★^*P* < 0.01 versus the Sil group; S: Sil group; X: Xfd group.

At the genus level, the microbiota composition greatly varied among the four groups (Figure 13). Compared with those in the Mod group, higher proportions of Lachnospiraceae_NK4A136_group and Candidatus_Saccharimoas were observed in the Sil and Xfd groups (*P* < 0.01, *P* < 0.05). Otherwise, compared with the Sil treatment, the Xfd treatment did not induce remarkable increases in abundance (*P* < 0.05) (Figures 14 and 15). A reduction in Muribaculaceae abundance was detected in the Sil group compared with that in the Mod and Xfd groups (*P* < 0.01). Xfd treatment did not induce a significantly reduced relative abundance compared to that in the Mod group (*P* < 0.01) (Figures 14–16).

### 3.5. Correlations between ALF-Related Factors and the Gut Microbiota

**Figure 17:**
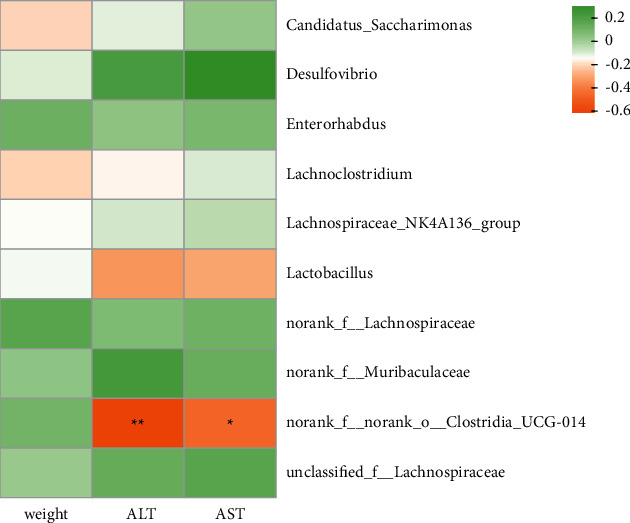
The relationship between ALF-related biochemical factors and the gut microbiota at the genus level. *Note.* The colors range from yellow (negative correlation) to green (positive correlation); statistical significance was based on ^★^*P* value < 0.05 or ^★★^*P* value < 0.01.

A correlation heatmap analysis was performed to investigate ALF-related factors and the significantly affected gut microflora at the genus level. We found that the ALT and AST levels were negatively correlated with Clostridia_UCG-014 abundance (*P* < 0.05 and *P* < 0.01) (Figure 17).

## 4. Discussion

ALF is a devastating clinical syndrome with high mortality and a substantial disease burden [21]. Although liver transplantation is the best treatment strategy for ALF, cadaveric organ shortages and subsequent complications limit this possibility. Multiple organ support and the use of antivirals and cell-based therapies demonstrate less encouraging efficacy; thus, great labor, material, and capital resources have been expended to identify alternative treatment methods for ALF in China [22].

Massive liver cell necrosis and extensive inflammation are the major changes in ALF [23, 24]. D-Gal/LPS can greatly stimulate inflammatory responses and increase endotoxin sensitivity and death in liver cells. An ALF model has been well established [14, 25]. From the perspective of TCM, “stasis” and “phlegm” define the key pathogenic features of ALF [26]. We used Xfd, which is a prescription that has been applied to treat ALF. Xfd regulates the liver, promotes blood circulation, strengthens the spleen, and resolves phlegm [27, 28].

BALB/*c* mice are widely used in animal immunology experiments. Since the weight change in the mice after 2 weeks was not very obvious, a significant difference was not observed. Moreover, Xfd had no effect on the weight of the liver. ALT and AST are major biochemical indicators of liver function. The ALT and AST levels in the Mod group were significantly increased compared with those in the Con group. Additionally, these elevations were rescued after Sil and Xfd pretreatment, indicating protective effects on ALF. The major histopathological changes in the livers of the mice after D-Gal/LPS exposure were the development of necrotic areas and severe hepatocyte necrosis. The liver appearance and texture associated with ALF were improved. Moreover, the Sil and Xfd treatments could also protect the liver tissue microstructure. The improvement in ALT and AST levels may be the most important reason.

These data clearly reveal that D-Gal/LPS-induced ALF was attenuated by Xfd treatment. We did not perform a high-performance liquid chromatography analysis of Xfd. This is a shortcoming of this study. We will perform this analysis in future research to obtain a clearer understanding of the main compounds present in Xfd. Tanshinone IIA is the primary active component extracted from *Salviae Miltiorrhizae Radix et Rhizoma.* Ma [29] reported pathological effects, revealing that tanshinone IIA remarkably mitigated pathologic damage to the liver, reducing hemorrhage and inflammatory cell infiltration. Consistent with the pathology results, tanshinone IIA improved ALT levels. Wang [30] demonstrated that tanshinone IIA pretreatment could protect the liver by activating the Nrf2 pathway. *Schisandrae Fructus Chinensis* prevented elevations in serum biochemical parameters, including AST and ALT levels. The mechanisms of *Schisandrae Fructus Chinensis* against drug-induced liver damage involve the regulation of inflammatory factors and oxidative stress [31]. Studies have suggested that *Schisandrae Fructus Chinensis* can accelerate the repair and regeneration of liver cells. In this experiment, this phenomenon was not observed due to the short time span from establishing the model to killing the mice. Saikosaponins, which are major bioactive compounds in *Bupleuri Radix*, possess anti-inflammatory and antiviral activities, regulate cell apoptosis, and have hepatoprotective effects, as described in a comprehensive review [32]. Furthermore, increased risks of overdose-induced histological injury in the mouse liver have also been reported and were involved in saikosaponin D-induced mitochondrial apoptosis in liver cells and potential hepatotoxicity [33]. *Citri Reticulatae Pericarpium* was shown to inhibit adipogenesis in 3T3-L1 preadipocytes. A marked reduction in PPAR-*γ*, C/EBP-*α*, and SREBP-1 levels played a positive role in protection against ALF [34]. *Mume Fructus* might play a therapeutic role in the improvement in ALT and AST levels by enhancing superoxide dismutase (SOD) activity and lowering malondialdehyde (MDA) content [35]. Accordingly, oxidative stress was improved in liver tissues, and hepatoprotective activities were observed [36].

Accumulating evidence indicates that multiple pathogenic factors that accelerate ALF are associated with gut microbiome imbalance [37–39]. Altered gut microbiota in terms of OTUs, the ACE diversity index, and PCA has been described by different groups. At the phylum level, Firmicutes and Bacteroidetes were the two main phyla of bacteria in the gut microbiota, and we found that the abundances of these two phyla were altered during D-Gal/LPS-induced ALF. An increased abundance of Firmicutes and a decreased abundance of Bacteroidetes have been widely reported to be associated with liver failure [40, 41]. There are conflicting conclusions regarding the abundances of Bacteroidetes and Firmicutes. For example, Yan and Hu reported that consumption of a high-fat diet caused an increased abundance of Bacteroidetes and a decreased abundance of Firmicutes [42, 43]. The abundance of Firmicutes was distinctly increased, and the abundance of Bacteroidetes and the Bacteroidetes/Firmicutes ratio were significantly decreased following Sil and Xfd treatment. However, the differences in Actinobacteriota and Desulfobacterota abundances were not significant due to the insufficient sample size. The fact that Xfd treatment reversed the gut microbiome imbalance may explain its hepatoprotective effects.

At the genus level, the microbiota composition greatly varied among the four groups. Currently, knowledge regarding the relationships between the members of Muribaculaceae and ALF is limited. Our data revealed that the decreased Muribaculaceae abundance might be a protective effect of Sil treatment. However, the effect of Xfd was not very obvious. Consistent with our results, Wang *G* found that *walnut green husk* polysaccharide consumption decreased the relative abundance of norank_f_Muribaculaceae at the genus level during a liver injury in Kunming male mice [44]. Polyene phosphatidylcholine is used to treat liver injury [45]. After polyene phosphatidylcholine administration, the relative abundance of Firmicutes also increased at the phylum level. Moreover, we found that the relative abundance of Lachnospiraceae_NK4A136_group was obviously elevated [46]. Surana demonstrated that an increased abundance of Lachnospiraceae_NK4A136_group was associated with anti-inflammatory effects [47]. Patients with ALF display evidence of systemic inflammatory response syndrome and local liver inflammation [48]. Huang provided evidence suggesting that the expression levels of IL-17*α* and TLR2 were negatively correlated with the abundance of Candidatus_Saccharimonas. In their research, Qingluo Tongbi decoction was shown to play a therapeutic role by decreasing the inflammatory responses regulated by the gut microbiota [49]. In our study, it was observed that the Sil and Xfd treatments can reduce the abundance of Candidatus_Saccharimonas, which may explain their protective effects against liver failure. We found a negative correlation between the abundance of Clostridia_UCG-014 and the levels of liver function biochemical indicators, especially ALT, suggesting the beneficial role of these bacteria in liver health. However, our knowledge regarding Clostridia_UCG-014 is very limited. Clostridia are thought to be short-chain fatty acid (SCFA) producers. SCFAs play an important role in human health based on the gut microbiota–host lipid metabolism axis [50, 51]. We sought to further investigate whether SCFA producers enriched by Sil and Xfd treatment prevent the progression of liver damage, but due to the lack of stool samples, this hypothesis was impossible to explore.

In conclusion, our findings demonstrate that Sil and Xfd treatment could notably alleviate D-Gal/LPS-induced ALF in mice, which might be correlated with decreased ALT and AST levels and reduced liver pathological damage. These protective effects may be mediated by alteration of the gut microbiota in ALF. Our findings provide new insight into the pretreatment of ALF and suggest that Xfd has probiotic potential against ALF and that its hepatoprotective effect on ALF patients needs to be proven in future clinical trials.

## Data Availability

The data used to support the findings of this study are included within the article.
